# Recurrent Clinical Burden and Cost of Cosmetic Surgery Tourism Complications: A Five-Year Retrospective Study From a UK Tertiary Centre

**DOI:** 10.7759/cureus.109006

**Published:** 2026-05-17

**Authors:** Pouya Mafi, Krishan Patel, Shuayb Hoque-Uddin, Robert Warner

**Affiliations:** 1 Plastic and Reconstructive Surgery, Queen Elizabeth Hospital Birmingham, Birmingham, GBR; 2 Plastic and Reconstructive Surgery, Birmingham Medical School, College of Medicine and Health, University of Birmingham, Birmingham, GBR

**Keywords:** cosmetic tourism, healthcare costs, health economics, medical tourism, nhs burden, plastic surgery complications, reconstructive surgery, resource utilisation, surgical complications

## Abstract

Introduction

Cosmetic surgery tourism can result in postoperative complications requiring management within publicly funded healthcare systems. This study aimed to evaluate the clinical and financial burden of complications following cosmetic surgery performed abroad at a UK tertiary centre, including repeat presentations and comprehensive resource utilisation.

Methods

A single-centre retrospective observational study was conducted at Queen Elizabeth Hospital, Birmingham, between 2020 and 2025. Patients presenting with complications following cosmetic procedures performed abroad within 12 months were identified through electronic patient record screening. Data were collected at the level of individual hospital presentations and included demographics, procedures performed, complications, healthcare utilisation, and cost. Cost analysis was performed using HRG4+ tariff mapping, the National Schedule of the National Health Service (NHS) Costs, and NHS Drug Tariff data. Descriptive statistical analysis was undertaken.

Results

A total of 749 patients were screened, yielding 29 patients accounting for 58 hospital presentations. Patients were predominantly female (96.6%) with a mean age of 42.3 years. A total of 61 procedures were performed abroad, most commonly in Turkey (82.8%). Common complications included wound infection, seroma, dehiscence, and necrosis, with venous thromboembolism also observed. The mean inpatient length of stay was 9.2 days, with 27 theatre interventions performed. Nearly half of all hospital presentations (48.3%) represented repeat presentations following an earlier complication-related attendance. The total cost across all presentations was £518,058.47, with a mean cost of £17,864 per patient. Cost per presentation ranged from approximately £46 to £57,691.

Conclusions

Complications following cosmetic surgery performed abroad impose a significant and recurrent burden on NHS services. When broader presentation-level costing and repeat presentations are considered, the financial impact appears greater than previously reported, although differences in costing methodology may partly contribute. These findings highlight the importance of improved patient awareness, informed decision-making, and systems-level measures such as enhanced patient information and more consistent data collection relating to cosmetic tourism complications.

## Introduction

Cosmetic surgery “tourism” (elective aesthetic procedures undertaken abroad, with patients returning home shortly afterwards) has become increasingly visible to UK acute services because postoperative complications frequently manifest after repatriation, when formal follow-up with the index surgeon is limited and operative documentation may be unavailable [1]. Contemporary UK evidence, largely from single-centre retrospective series, consistently demonstrates clinically significant morbidity, including wound infection/dehiscence, necrosis, seroma/haematoma, implant-related complications, and thromboembolic events requiring antibiotics, reoperation, prolonged inpatient stay, and outpatient/community aftercare [2-4].

Global demand for aesthetic procedures continues to rise. The International Society of Aesthetic Plastic Surgery (ISAPS) reported more than 33.8 million surgical and non-surgical aesthetic procedures worldwide in 2022 [5]. Cost differentials, online marketing, and “surgery-plus-travel” package models continue to drive overseas cosmetic surgery, with complications frequently presenting to the National Health Service (NHS) during the early postoperative period after return to the UK [1]. However, the true scale of cosmetic surgery tourism remains difficult to quantify because routine datasets do not systematically capture surgery performed abroad. A recent rapid review cited estimates suggesting at least 348,000 UK residents travelled abroad for medical treatment in 2022, although this figure was not specific to cosmetic surgery [1,6]. Despite uncertainty regarding denominator data, multiple UK tertiary centres continue to report substantial downstream burden associated with postoperative complications [3,7].

UK guidance emphasises that the NHS provides emergency treatment for serious postoperative complications, while elective revision for unsatisfactory cosmetic outcomes is generally not commissioned [8]. The British Association of Plastic, Reconstructive and Aesthetic Surgeons (BAPRAS) has also highlighted concerns regarding patient awareness, regulation, and continuity of care through sustained public safety campaigns [9]. Across UK series, complications commonly require intravenous antibiotics, operative washout/debridement, negative-pressure wound therapy, explantation procedures, or prolonged reconstructive follow-up [1-3,10-12]. Thromboembolic complications represent an additional travel-related risk, particularly where early repatriation follows major surgery [7,13]. Plastic surgery literature suggests that cosmetic tourism-related presentations continued during pandemic restrictions and persisted as international travel resumed [14]. This strengthens the rationale for updated post-COVID analyses rather than reliance on pre-pandemic cohorts alone.

Queen Elizabeth Hospital Birmingham is a major UK tertiary referral and trauma centre with specialist reconstructive surgical services, making it a plausible receiving centre for severe complications following cosmetic surgery abroad. A previous local study evaluating presentations between 2015 and 2020 estimated a total cost of £152,946 (mean £5,882.54/patient) using the National Schedule of NHS Costs [2]. However, that analysis predated the post-COVID travel rebound and used a narrower costing approach primarily focused on inpatient admissions, without comprehensive inclusion of emergency department attendances, outpatient follow-up, imaging, medication costs, critical care utilisation and repeat presentations, all of which were incorporated into the present analysis. More recent UK cohorts suggest the burden may be substantial and potentially rising, with a Scottish series reporting costs exceeding £755k across 81 patients between 2019 and 2023 [3]. The 2026 rapid review concluded that evidence regarding NHS financial burden remains fragmented and called for more systematic approaches to costing and data collection [1].

This retrospective observational study evaluates the acute and downstream resource burden of complications from cosmetic procedures performed abroad, presenting to Queen Elizabeth Hospital Birmingham between 2020 and 2025. It specifically focuses on the post-COVID period, recurrent presentations, and broader whole-pathway costing. The objectives were to (1) describe patient characteristics, procedures performed abroad, and time-to-presentation; (2) characterise complications, severity, repeat presentation, and multidisciplinary involvement; (3) quantify NHS resource utilisation across emergency, inpatient, operative, imaging, outpatient, and medication-related care; and (4) estimate costs using contemporary national costing sources.

## Materials and methods

Study design

This was a single-centre retrospective observational study conducted at the Department of Plastic Surgery, Queen Elizabeth Hospital, Birmingham, United Kingdom, a tertiary major trauma centre. The study aimed to quantify the clinical and financial burden associated with complications following cosmetic procedures performed abroad over the period from January 2020 to December 2025. Patients were identified through interrogation of the hospital’s electronic patient record system using a predefined coding field consisting of a mandatory tick-box indicating hospital admission abroad within the preceding 12 months. This search yielded 749 patients, who were subsequently manually screened to identify those admitted abroad for cosmetic procedures and assessed against predefined inclusion and exclusion criteria.

Inclusion criteria

Patients were included if they were aged 18 years or older and had undergone cosmetic surgery performed outside the UK. Eligible patients were those who subsequently presented with complications requiring hospital-based management, including emergency department assessment, inpatient admission, operative intervention, specialist inpatient review, or outpatient plastic surgery follow-up. Only patients presenting within 12 months of their procedure abroad were included in the analysis.

Exclusion criteria

Patients were excluded if they had undergone non-cosmetic procedures or if their procedure had been performed within the UK. Patients who presented with complications more than 12 months after undergoing cosmetic surgery abroad were also excluded.

Data collection

Following the application of the inclusion and exclusion criteria, the final cohort comprised 29 unique patients, accounting for 58 presentations to the hospital. Data were collected at the level of individual presentation. Variables recorded included patient demographics, smoking status, comorbidities, country of procedure, type and number of procedures performed, route of presentation, complication type, level of care required, multidisciplinary team involvement, recurrence of complications, readmission status, length of stay, theatre interventions, imaging, laboratory and microbiological investigations, outpatient follow-up, medication use, and overall healthcare utilisation. Recurrence was defined as repeat presentation or readmission related to the same index overseas cosmetic procedure complication following the initial episode of care.

Costing methodology

Cost analysis was performed at the level of individual admissions using a combination of the National Schedule of NHS Costs, HRG4+ tariff mapping, and NHS Business Services Authority Drug Tariff data. Cost components included emergency department attendance, inpatient bed days, critical care utilisation, investigations and imaging, operative procedures, outpatient follow-up, and medication costs.

For admissions involving multiple theatre episodes within a single inpatient spell, the highest-complexity procedure was costed using the non-elective long-stay HRG tariff. Additional procedures within the same spell were costed using the non-elective short-stay tariff as a proxy for incremental theatre resource utilisation. This approach was selected to better reflect actual resource consumption, while acknowledging the potential for slight overestimation compared with standard HRG grouping for reimbursement purposes.

Statistical analysis

Descriptive statistical analysis was performed. Categorical variables are presented as frequencies and percentages. Continuous variables are summarised using mean, median, and range, as appropriate. No inferential statistical testing was undertaken, as the primary aim of the study was to characterise the burden and resource utilisation associated with these presentations.

## Results

A total of 749 patients were screened using the electronic patient record system for a history of hospital admission abroad. Following application of inclusion and exclusion criteria, 29 patients were identified, accounting for 58 presentations related to complications following cosmetic surgery performed outside the United Kingdom. Patients were predominantly female (96.6%), with an age range of 20-59 years, a mean age of 42.3 years, and a median age of 41 years. Table 1 demonstrates the patient distribution by demographics. Ethnically, the cohort was diverse, with representation across White, Asian, and mixed ethnic groups (Table 2).

**Table 1:** Patient demographics (n = 29)

Demographics	Value
Number of patients	29
Total presentations	58
Female sex	28 (96.6%)
Age range (years)	20–59
Mean age (years)	42.3
Median age (years)	41

**Table 2:** Ethnicity distribution

Ethnicity	n
White British	11
Asian British – Pakistani	6
Asian British – Indian	1
Asian British – Other	5
White Other	3
Mixed	3

All index procedures were performed overseas. Turkey was the most common destination, accounting for 24 patients (82.8%), with the remainder distributed across multiple countries. Table 3 summarises the distribution of procedures by country. A total of 61 procedures were performed across 29 patients, with several patients undergoing multiple procedures within a single operative episode. Table 4 provides a breakdown of all procedures performed by type of intervention and their frequency.

**Table 3:** Country of procedure

Country	n (%)
Turkey	24 (82.8%)
USA	1 (3.4%)
Lithuania	1 (3.4%)
Cyprus	1 (3.4%)
Brazil	1 (3.4%)
Pakistan	1 (3.4%)

**Table 4:** Types of procedures performed abroad (total procedures n = 61) *Hernia repair was performed concurrently with cosmetic surgery during the same overseas operative episode.

Procedure	n
Abdominoplasty	22
Breast augmentation/implant	13
Liposuction	11
Breast reduction/lift	5
Thigh lift	3
Monsplasty	2
Rhinoplasty	1
Buttock augmentation	1
Brachioplasty	1
Deltoid implants	1
Hernia repair (same episode)*	1

Complications were heterogeneous and frequently multifactorial, with multiple complications occurring within a single presentation. The most common complications were wound infection and seroma, followed by wound dehiscence and skin or fat necrosis. More severe complications included implant-related complications, venous thromboembolism, and sepsis (Table 5).

**Table 5:** Complications per admission (n = 58, multiple per admission possible) *Other includes cellulitis without wound infection, dehydration, inadequate pain management, equipment-related issues (e.g. VAC), graft-related complications, suture-related problems, delayed wound healing, and non-specific postoperative symptoms. DVT: Deep Vein Thrombosis; PE: Pulmonary Embolism; VAC: Vacuum-Assisted Closure

Complication	n
Wound infection	22
Seroma	20
Other*	19
Wound dehiscence	14
Skin/fat necrosis	11
Implant complication	6
Venous thromboembolism (DVT/PE)	5
Abscess	3
Haematoma	3
Sepsis	2

The emergency department was the most common route of presentation, accounting for 33 admissions (57%). A proportion of patients were referred via primary care or transferred from other hospitals. Table 6 summarises the different routes of presentation. Ward-only presentations refer to direct presentations to specialist services without an emergency department assessment. The majority of presentations required inpatient care, with a small number requiring escalation to high-dependency or intensive care (Table 7).

**Table 6:** Route of presentation

Route	n (%)
Emergency department	33 (57%)
GP referral	7 (12%)
Inter-hospital transfer	3 (5%)
Other	15 (26%)

**Table 7:** Level of care ED: Emergency Department, ITU: Intensive Treatment Unit, HDU: High-Dependency Unit

Level of care	n (%)
Ward only	31 (53.4%)
Ward + ED	18 (31.0%)
ED only	7 (12.1%)
ITU/HDU	2 (3.4%)

Patients demonstrated significant healthcare utilisation. The mean inpatient length of stay for admitted presentations was 9.2 days (median 6 days, range 1-41 days). A total of 27 theatre visits were recorded across the cohort. Nearly half of the presentations (48.3%) were associated with recurrent complications requiring further healthcare contact. Table 8 provides a summary of healthcare utilisation. A wide range of imaging modalities was utilised, including ultrasound, computed tomography, chest radiography, Doppler studies, and magnetic resonance imaging.

**Table 8:** Healthcare utilisation summary

Variable	Value
Total presentations	58
Mean length of stay (days)	9.2
Median length of stay (days)	6
Range length of stay (days)	1–41
Total theatre visits	27
Repeat presentation rate	48.3%

The principal drivers of overall cost were inpatient bed utilisation, operative intervention, and critical care admission, where required. The total cost incurred across all presentations was £518,058.47, equating to a mean cost of £17,864 per patient. The cost per presentation demonstrated marked variability, ranging from approximately £46 to £57,691, reflecting the heterogeneity in complication severity and required interventions. The cost analysis is summarised in Table 9. Lower-cost presentations were typically limited to brief assessment episodes requiring minimal investigation or intervention without inpatient admission, whereas higher-cost presentations were driven by prolonged inpatient stays, operative intervention, and reconstructive procedures.

**Table 9:** Cost analysis summary

Variable	Value
Total cost (all presentations)	£518,058.47
Mean cost per patient	£17,864
Cost per presentation (range)	£46–£57,691
Number of presentations	58
Number of patients	29

## Discussion

This study demonstrates a substantial ongoing burden of complications following cosmetic surgery performed abroad at a UK tertiary major trauma centre. Over a five-year period, a relatively small cohort of 29 patients accounted for 58 presentations, generating a total cost exceeding £500,000. These findings highlight that the burden of cosmetic tourism complications is not limited to the initial presentation but is driven by recurrent and resource-intensive healthcare utilisation [1,7].

Previous studies have reported a measurable burden associated with cosmetic tourism on publicly funded healthcare systems [1-4]. A prior study from the same institution reported a total cost of £152,946 across 26 patients, with a mean cost of approximately £5,882 per patient. In contrast, the present study demonstrates a mean cost of £17,864 per patient, representing a near threefold increase. This discrepancy likely reflects a combination of factors, including differences in costing methodology and increasing clinical complexity. In the present study, presentation-level costing was used, allowing the capture of multiple presentations per patient, whereas earlier work primarily reported patient-level cost. Furthermore, a more comprehensive costing approach was employed, incorporating HRG-based tariffs, medications, imaging, and post-discharge care. These findings likely reflect both increasing healthcare utilisation and important methodological differences between studies, limiting direct comparison. While the present analysis incorporated broader presentation-level costing and repeat presentations, aspects of this methodology may also overestimate certain cost elements in complex admissions.

A key finding of this study is the marked variability in cost per presentation, ranging from approximately £46 to over £57,000. This reflects the heterogeneous nature of complications arising from cosmetic procedures performed abroad. Lower-cost presentations were generally associated with short-stay or conservatively managed presentations, whereas higher costs were driven by prolonged inpatient stays, operative management, and reconstructive procedures. This variation highlights that while some complications may be minor, a subset of presentations requires prolonged inpatient care, operative intervention, and critical care support, contributing disproportionately to overall healthcare expenditure.

Nearly half of the presentations in this cohort were associated with repeat complications or readmission. This finding is particularly important, as it challenges the assumption that complications following cosmetic tourism represent isolated events. Instead, these patients frequently require repeated hospital visits, ongoing wound care, further surgical intervention, and prolonged outpatient and community follow-up. This pattern of repeat presentation significantly amplifies the cumulative burden on NHS services and represents a key driver of overall cost [7,10,11].

A substantial proportion of patients in this study required operative management, including debridement, negative-pressure wound therapy, and reconstructive procedures such as split-thickness skin grafting. These interventions are typically reserved for complex wound pathology and reflect the severity of complications encountered [12]. In addition, patients frequently required input from multiple specialities, including microbiology, radiology, physiotherapy, and community care services, further increasing the complexity and cost of management [7,10]. A small but important subset of patients required escalation to high-dependency or intensive care, demonstrating that complications following cosmetic surgery abroad can extend beyond local wound issues and may represent significant systemic illness requiring high-acuity care [1,8].

The present cohort included several complex and resource-intensive presentations, including prolonged admissions, operative intervention, and critical care utilisation. This may reflect evolving trends in cosmetic tourism, including the increasing prevalence of multiple concurrent procedures, shorter recovery periods prior to return travel, and limited access to structured postoperative follow-up [7,14]. Differences in perioperative pathways, travel timing, postoperative follow-up access, and patient-related factors may contribute to complications such as necrosis, infection, and thromboembolic events observed in this cohort [12,13].

These findings have important implications for healthcare systems. Complications arising from cosmetic surgery performed abroad represent an important systems-level burden for publicly funded healthcare services, as complications arising after privately funded overseas procedures frequently require NHS management [7,8]. Despite the elective nature of the index procedures, management of complications is frequently complex, prolonged, and resource-intensive. The cumulative cost demonstrated in this study likely underestimates the true burden, as it does not fully capture primary care involvement, all aspects of community nursing, psychological impact, and indirect societal costs such as loss of productivity [1,7].

This study has several strengths. The use of granular, admission-level costing incorporating HRG-based tariffs, medication costs, and hospital-based follow-up care allowed for a more comprehensive estimation of healthcare utilisation than has been reported previously [1]. Additionally, the inclusion of repeat presentations provides insight into the longitudinal burden of complications, which has been underrepresented in existing literature [7].

However, several limitations should be acknowledged. Case identification relied on a database search for patients with documented hospital admission abroad, which may not capture all individuals undergoing cosmetic procedures overseas [6]. Furthermore, restricting inclusion to presentations within 12 months of the index procedure may exclude delayed complications [14]. Cost estimation may represent overestimation in cases where multiple procedures within a single presentation were costed individually rather than grouped under a single HRG episode. Conversely, some aspects of care, particularly community-based primary care utilisation and indirect social costs, were not fully captured, suggesting that the overall burden may still be underestimated [1,7]. As a single-centre study, findings may not be fully generalisable, although patterns observed are likely reflective of other tertiary centres managing similar patient populations [10,11]. Additionally, the tertiary referral and major trauma centre status of the institution may have influenced the severity and complexity of presentations captured, potentially increasing observed healthcare utilisation and associated costs compared with non-tertiary settings.

## Conclusions

Complications following cosmetic surgery performed abroad impose a significant and recurrent burden on NHS services. When broader presentation-level costing and repeat presentations are considered, the estimated financial impact appears greater than previously reported, although differences in costing methodology and resource capture may partly contribute to this finding. These findings highlight the importance of improving patient awareness and informed decision-making regarding overseas cosmetic surgery, alongside consideration of systems-level measures, such as enhanced patient information and more consistent data collection relating to cosmetic tourism complications within publicly funded healthcare systems.
